# Supplementary figures and images for: In silico mutagenesis of human ACE2 with S protein and translational efficiency explain SARS-CoV-2 infectivity in different species
Source: PLoS Comput Biol. 2020 Dec 7;16(12):e1008450. doi: 10.1371/journal.pcbi.1008450 (PMC7746295; doi:10.1371/journal.pcbi.1008450)

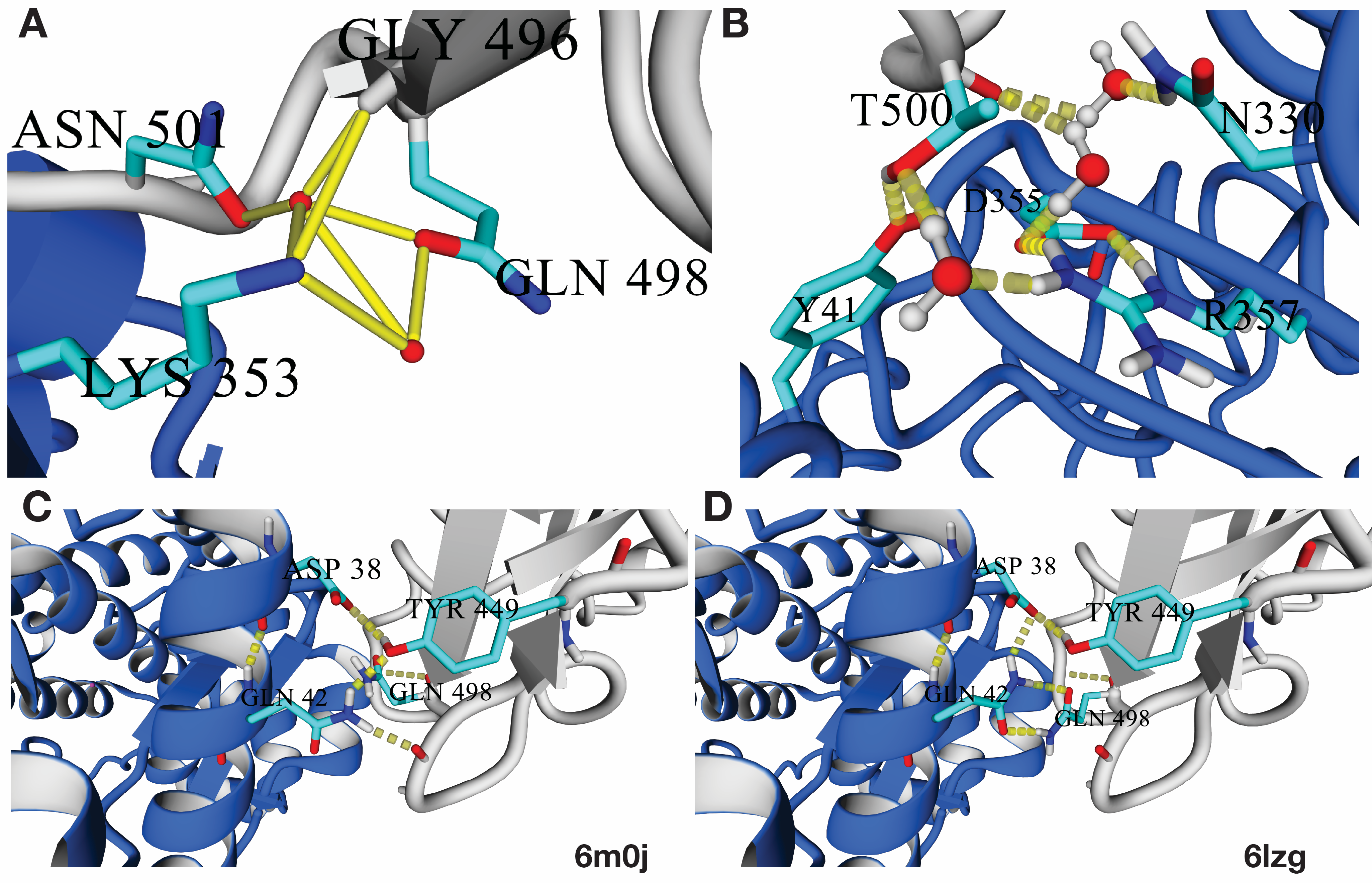

Supplement: S1 Fig — (A) Water bridge between hACE2-K353, S-N501, S-Q498, S-G496 (B) Hbond network and water bridges for hACE2-R357. (C) Hbond network around D38 in the 6moj structure. (D) Hbond network around D38 in the 6lzg structure, it can be seen how the O of the OH group of Tyr449 is at H-bond distance to the CO group of Gln 498 which is not possible. (TIF) [file pcbi.1008450.s001.tif]

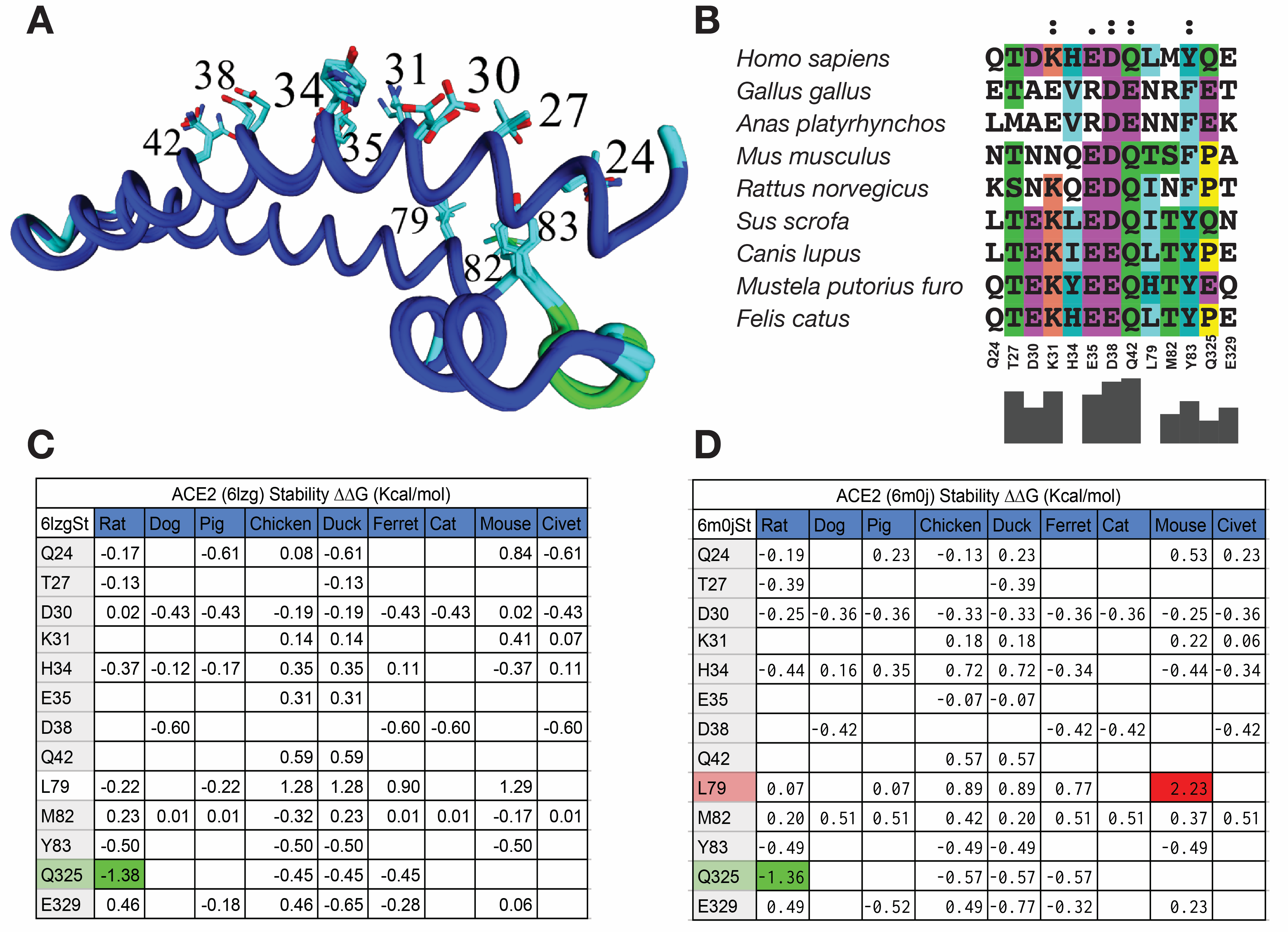

Supplement: S2 Fig — (A) Superimposition of the three alpha-helices (24–53,54–83,90–103) contacting the S protein from all X-ray structures (6lzg, 6m0j, 6vw1-2 crystallographic dimers, 3doi-2 crystallographic dimers). (B) Local sequence alignment of the ACE2 residues that are in the region of the ACE2 that contacts against the S protein. We show those that are different between the species. (C) Changes in folding stability of the hACE2 protein upon single point mutations from human to the animal species using either the 6lzg or 6moj structures. (TIF) [file pcbi.1008450.s002.tif]
