# Supplementary material for: In silico mutagenesis of human ACE2 with S protein and translational efficiency explain SARS-CoV-2 infectivity in different species
Source: PLoS Comput Biol. 2020 Dec 7;16(12):e1008450. doi: 10.1371/journal.pcbi.1008450 (PMC7746295; doi:10.1371/journal.pcbi.1008450)
Supplement: S1 Text — It includes the commands used for running repair PDB, ACE2/S interaction energy, ACE2 stability, and water prediction. (PDF) [file pcbi.1008450.s009.pdf]

FOLDX COMMANDS USED FOR CALCULATIONS:

Before running FoldX please look at the documentation included on the site:

http://foldxsuite.crg.eu/documentation

FoldX Repair PDB:

First of all 6m0j, 6lzg, 6vw1 pdb files were repaired with FoldX to fix crystallographic errors using RepairPDB command (http://foldxsuite.crg.eu/command/RepairPDB), and in the case of 6vw1 previously splitted into two pdb files, one for each dimer (6vw1_1,6vw1_2):

>foldx --command=RepairPdb --pdb=pdbFile.pdb

ACE2/S1 Interaction Energy:

Interaction ΔΔG upon mutation with FoldX is calculated in two steps, first run BuildModel command (http://foldxsuite.crg.eu/command/BuildModel) over the repaired files:

>foldx --pdb=pdbRepired.pdb --command=BuildModel --mutant-file=individual_list.txt

Then run AnalyseComplex command (http://foldxsuite.crg.eu/command/AnalyseComplex) with the labels required for water prediction (*--pdbWaters=true --water=-PREDICT*) for BuildModel output pdb files:

>foldx --pdb=BuildModelOutputFile.pdb --command=AnalyseComplex --analyseComplexChains=A,E --pdbWaters=true --water=-PREDICT

Then we subtract the interaction energies from the AnalyseComplex output files:

$$\Delta\Delta G=\Delta G\_MT-\Delta G\_WT$$

ACE2 Stability:

In order to calculate ACE2 Stability ΔΔG upon mutations, S1 protein molecules were erased from the ACE2/S1 pdb files, resultant models were repaired with RepairPDB and BuildModel

command was run to calculate the effect of mutations. ΔΔG was directly extracted from the output files.

Water Prediction:

Water prediction with CrystalWaters command (http://foldxsuite.crg.eu/command/CrystalWaters):

>foldx --command=CrystalWaters --pdb=pdbFile.pdb --water=-PREDICT --pdbWaters=true --out-pdb=true --pdbHydrogens=true
