# Supplementary material for: In silico mutagenesis of human ACE2 with S protein and translational efficiency explain SARS-CoV-2 infectivity in different species
Source: PLoS Comput Biol. 2020 Dec 7;16(12):e1008450. doi: 10.1371/journal.pcbi.1008450 (PMC7746295; doi:10.1371/journal.pcbi.1008450)
Supplement: Attachment — Submitted filename: Blanco_Serrano-PlosCB_review-20200518.pdf [file pcbi.1008450.s010.pdf]

In this manuscript, the authors focus on an important question using assorted bioinformatics tools – how do corona spike proteins interact with their host ACE2 receptors, and how these interactions and additional processes, such as translation efficiency, might affect viral adaptation. This is a worthwhile goal, especially at this time, having relevance for understanding these interactions and implications for addressing the current pandemic.

However, the manuscript requires addressing several major issues:

1. The introduction requires much improvement – it should be expanded and updated to include an appropriate coverage of the relevant literature at this point in time. Given how quickly the relevant literature is evolving, I strongly recommend stating the current time point at the end of the revision (e.g. "as of May 2020").

Some of the relevant structural papers are not cited (see also below). It is also important to clarify previous research on SARS-CoV-1 or MERS vs. studies on the current SARS-CoV-2, emphasizing what is known about the different viruses/spike proteins and their common/different structural and sequence characteristics. Such critical issues are not covered clearly nor comprehensively.

2. The results of the structural analysis are not analyzed critically. In particular, the authors did not present their full results for several parts of the results. Appropriate controls or comparisons should be made at every section to provide the reader with a better understanding of the strengths and limitations of the analysis (see main examples below).

3. The first part of the results is misleadingly called "structural analysis of the ACE2-S protein complex", while in fact it is a prediction of water molecules that are absent in selected structures the authors chose to analyze. I agree an analysis of waters can lead to new insights, but at the very least the authors need to apply it to all available structures and report the results in full and not in a qualitative and low resolution way for a selected (based on what exactly?) single structure, as in Fig. 1. For example, Fig. 1D does not really show in "atomic detail" the water networks. I believe it is critical to show which of these waters are predicted similarly in the majority of all comparable structures (including the Cov-1 structures, but clearly stating which is which).

4. Recent papers have shown the assumptions made by the authors regarding infectivity in different species might be premature (e.g. dogs do seem to be susceptible). Better to analyze the bioinformatic data without assumptions, and leave the comparison to the literature for the Discussion.

5. The in silico mutagenesis part is indeed important, but lacking. Why show as the main result here (Fig. 2A) a ddG per species? Much more interesting and useful to the community to show a per-residue plot for each species, aligned. Similarly, a full sequence alignment should be shown, rather that showing only selected residues (Fig. 2B).

6. Certainly the authors can find much more detailed (cell-specific?) expression data to compare against their translation efficiency analysis? By now transcriptomic data is far from “scarce”.

7. A critical analysis is missing – do mutate residues all along the spike protein to all possible residues, plot the predicted ddG values for stability and for the effect on interactions with ACE2, and also compare to known data on mutations in the spike protein(s) (i.e. why focus on ACE2 only). Even if prediction accuracy is far from perfect, such computational predictions can help scientists hypothesize what are the outcomes of mutations that are already being detected in the spike protein across the globe or that might be identified in the future.(No, Table S3 does not address this point at all.)

8. The discussion should also point where are the uncertainties in the predictions, and the known / predicted shortcomings of these predictions.

9. A minor point – the M&M section should point to a supplementary document listing the full commands used to run the calculations. The current text is not sufficient to allow the reader to repeat the calculations as they were performed.
