# Supplementary material for: In silico mutagenesis of human ACE2 with S protein and translational efficiency explain SARS-CoV-2 infectivity in different species
Source: PLoS Comput Biol. 2020 Dec 7;16(12):e1008450. doi: 10.1371/journal.pcbi.1008450 (PMC7746295; doi:10.1371/journal.pcbi.1008450)
Supplement: Attachment — Submitted filename: Response_Letter.pdf [file pcbi.1008450.s011.pdf]

Dear Dr. Serrano,

Thank you very much for submitting your manuscript "ACE2 genetic variability and codon usage explains coronavirus infectivity in species suggesting different resistance degrees in humans" for consideration at PLOS Computational Biology.

As with all papers reviewed by the journal, your manuscript was reviewed by members of the editorial board and by several independent reviewers. In light of the reviews (below this email), we would like to invite the resubmission of a significantly-revised version that takes into account the reviewers' comments.

We cannot make any decision about publication until we have seen the revised manuscript and your response to the reviewers' comments. Your revised manuscript is also likely to be sent to reviewers for further evaluation.

When you are ready to resubmit, please upload the following:

[1] A letter containing a detailed list of your responses to the review comments and a description of the changes you have made in the manuscript. Please note while forming your response, if your article is accepted, you may have the opportunity to make the peer review history publicly available. The record will include editor decision letters (with reviews) and your responses to reviewer comments. If eligible, we will contact you to opt in or out.

[2] Two versions of the revised manuscript: one with either highlights or tracked changes denoting where the text has been changed; the other a clean version (uploaded as the manuscript file).

Important additional instructions are given below your reviewer comments.

Please prepare and submit your revised manuscript within 60 days. If you anticipate any delay, please let us know the expected resubmission date by replying to this email. Please note that revised manuscripts received after the 60-day due date may require evaluation and peer review similar to newly submitted manuscripts.

Thank you again for your submission. We hope that our editorial process has been constructive so far, and we welcome your feedback at any time. Please don't hesitate to contact us if you have any questions or comments.

Sincerely,

Rachel Kolodny
Guest Editor

PLOS Computational Biology

Nir Ben-Tal
Deputy Editor
PLOS Computational Biology

****

Reviewer's Responses to Questions

Comments to the Authors:
Please note here if the review is uploaded as an attachment.

Reviewer #1: Title: ACE2 genetic variability and codon usage explains coronavirus infectivity in species suggesting different resistance degrees in humans

Summary: The authors investigate the infectivity of SARS-CoV-2 through computational analysis of several different topics. They begin with modeling the human ACE2 – SARS-CoV-2 S protein complex using the software FoldX in order to predict the presence of water bridges that could contribute to stronger binding between the two molecules. They proceed to model the same complex using amino acid residues from homologues ACE2 in other potential host animals at the ACE2 – S complex interface. They compare the codon adaptation index of the viral genes in human and animals as well as the lung tissue-specific expression of ACE2 in each system. They end with an analysis of 229 reported SNPs in the ACE2 receptor and their potential impact on the binding of the ACE2 – S protein complex. While the manuscript provides some interesting modeling, the different analyses that are conducted are not inherently linked and the conclusions that are drawn are not well supported. In order to be considered for publication, the study should unify the message of these varied investigations, either increasing focus on ACE2 – S protein interactions with a more robust computational analysis or considering other host interactions with the viral S protein.

We offer the following comments:

1. As the manuscript does not go into extensive detail regarding the ACE2 – S protein interaction beyond what has heretofore been published, the authors could expand their analysis beyond viral protein interactions with the ACE2 receptor. Zhou et al. (2020) find 119 host proteins associated with coronaviruses, including SARS-CoV-2. If the authors intend to include an analysis of other

species and their ACE2 receptors, they should consider expanding the analysis to include the known interactome.

Response to point 1:

We thank the reviewer for the suggestion but unfortunately the known interactomes for SARS-CoV-2 are lacking high resolution structures for complexes between viral and human proteins. The only complex available is that of the S1 domain and human ACE2. This is also an important one since it constitutes the entry point for the virus. Our experience tells us that most homology modeling and docking methods are good to predict topologies and binding surfaces. However, they are not good to predict sidechain packing with accuracy which is necessary for proper evaluation of mutations. Even with crystal structures good resolution and quality is important for accurate predictions.

2. The authors analyze the binding energies between the SARS-CoV-2 S protein and ACE2 receptor in human and other animals. They rely on the published crystal structure of human ACE2 – S protein complex and introduce amino acid mutations where the animal residues are different at the interface of the complex. This might be sufficient but docking (with a tool such as ZDOCK) is more realistic. Docking may find that this orientation isn't achievable due to clashing in intermediate orientations in the docking process. Furthermore, the assumption that ACE2 in other animals will have the same structure is not well based. Other parameters should be taken in consideration such as conservation, mRNA structure, etc.

Response to point 2:

The referee is correct, we didn't explain in detail the reason for going ahead with the analysis without explaining why we decided to rely on the released crystal structures. In this new version we now included the alignment for all species considered here. The alignment shows high sequence similarity for the ACE2 regions which is responsible for the binding to SARS-CoV-2 S protein. We also did a docking exercise with the ACE2 and S proteins taken separately from the complex using ZDOCK. We found that for 2 out of the 10 models ZDOCK found the correct binding region but these two models presented too many Van der Waals' clashes that made them not suitable for protein design.

To see if the aa differences in the region of ACE2 interacting with the RBD domain of the S protein could change the conformation of this region we performed a stability study for the animal sequence variations. The idea is that if there is a mutation that indicates strong destabilization (>1.5kcal/mol) we could expect a conformational change. We find that, with one exception at position 97 for some species, the amino acid differences between species do not

significantly destabilize the structure and can be readily accommodated in the crystal structure of human ACE2. Regarding position 97, it is solvent exposed and therefore it could be accommodated. Moreover structural superimposition of the civet ACE2 interface with the human one shows they are practically identical within the crystallographic error (S1 Figure and S5 Table). We have also modified Figure 2 to include the alignment of the binding region for all species included in the manuscript.

3. The authors relate the infectivity of SARS-CoV-2 to the CAI of its genes in human and other potential hosts. They also attempt to draw a connection between ACE2 expression in host lung tissue and infectivity. It is noted that SARS-CoV-2 infects cats, yet the authors report some of the lowest CAIs for viral genes in Felis catus. They also report one of the lowest ACE2 expression levels in human lung. These contradictory analyses do not add to the message of the manuscript. CAI is a metric of codon usage bias and is not directly linked to translation efficiency. Additional approaches should be taken to estimate translation efficiency, otherwise this part should be eliminated altogether. The low expression of ACE2 in human lung and high expression in mouse lung (which is not infected by SARS-CoV-2) is not explained in the context of the study. In general, this is the weakest part of the study, it does not have novelty, while the conclusions are only loosely supported by the data.

Response to point 3:

As suggested by the reviewer, we have now incorporated another well-established estimate of translational efficiency: the tRNA adaptation index (tAI). Rather than measuring the codon bias with regard to the host, the tAI shows the correspondence between the codon usage and the tRNA levels of the host (approximated by the tRNA gene copy numbers). Despite few differences, the tAI shows a very similar profile of adaptation across species (Fig. 3A-B). With this additional evidence, we believe that the effect of translational efficiency in determining viral infectivity is now more solid and stronger. Regarding the comments on the ACE2 gene expression, the data shown here indicates that low levels of ACE2 are sufficient to cause infection, as far as the virus has an optimal interaction with the spike and is translationally adapted. In fact, *Homo sapiens* is the species with both the highest tAI and the highest interaction, which could explain the high transmissibility of the virus among humans.

4. The authors analyze 229 mutations in the human ACE2 receptor. While they utilize the Single Nucleotide Polymorphism Database (dbSNP), they could improve the power of this analysis by including data from NCBI's database of human genomic structural variation (dbVAR).
In its current state, the manuscript does not gain from analysis of CAI and ACE2 expression in other species. These investigations could be interesting if made more thorough. Currently the authors offer evidence that is contradictory to the message of the study. It is recommended that

the authors either expand these analyses with more robust metrics and experimental data if these are available or instead focus on the ACE2 – S protein interaction in human, including other proteins from the interactome and a more comprehensive analysis of variation in human ACE2.

Response to point 4:
Thanks to the referee for pointing out this issue, there was a mistake in the paper because we extracted human variants from Ensembl that compiles gnomAD, TopMed, ExAc, ESP, 1000Genomes. Afterwards we filter for missense mutations. We did not look at changes in exons or deletions/insertions since this will require more complex modeling which as indicated for docking will probably not be very reliable. Following point 4 suggestion, we have included 31 dbVar missense mutations that were retrieved on the initial manuscript (S3 Table), no one of them showed significant changes in affinity, while some of them that are not involved in the interaction have significant changes in ACE2 stability.

Reviewer #2: uploaded as an attachment

In this manuscript, the authors focus on an important question using assorted bioinformatics tools – how do corona spike proteins interact with their host ACE2 receptors, and how these interactions and additional processes, such as translation efficiency, might affect viral adaptation. This is a worthwhile goal, especially at this time, having relevance for understanding these interactions and implications for addressing the current pandemic.

However, the manuscript requires addressing several major issues:

1. The introduction requires much improvement – it should be expanded and updated to include an appropriate coverage of the relevant literature at this point in time. Given how quickly the relevant literature is evolving, I strongly recommend stating the current time point at the end of the revision (e.g. “as of May 2020”).

Some of the relevant structural papers are not cited (see also below). It is also important to clarify previous research on SARS - CoV - 1 or MERS vs. studies on the current SARS - CoV - 2, emphasizing what is known about the different viruses/spike proteins and their common/different structural and sequence characteristics. Such critical issues are not covered clearly nor comprehensively.

We followed recommendations of the referee and apart from updating the literature we have made a thorough analysis of the most relevant crystal structures deposited in the protein data bank (PDB). Given the urgency this pandemic is causing we could have omitted this careful

analysis so we thank the reviewer for pointing us in that direction. We were very surprised that some models with similar resolution don't have enough quality according to PDB sanity parameters (ramachandran, sidechain and RSRZ outliers) and FoldX energies, while others have suspiciously similar PDB coordinates that suggest that they were not assigned but created from other existing models. We have included a supplementary table (S5 Table) reporting quality parameters and per object RMSDs for the most important ACE2/SARS-CoV-2 S protein complexes. The best model according to these quality parameters and FoldX stability is 6m0j, which was not included in the previous version of the manuscript. For this reason we have redone the same analysis with a new ACE2/SARS-CoV-2 S protein crystal structure (6m0j) that has passed all quality control filters and discarded 6vw1 which in fact is a chimaera and has low quality PDB parameters and bad packing energy compared to 6lzg and 6m0j. We have found, after looking carefully at 6lgz, that there is an intramolecular glutamine/asparagine network where the orientation of the side chain CO and NH2 groups are incorrectly placed (S2A-B Figure) which interacts with D38 of ACE2. This results in an incorrect prediction on binding energy when mutating D38 to Glu (see new Discussion). This network is in the correct orientation in 6m0j. Prediction of the binding energies in different species using 6m0j results in an excellent agreement with the experimental evidence (see new Figure 2). Changing the incorrect Gln network in 6lzg also results in the correct prediction of D38E mutation. We have included a block explaining the selection of the crystallographic models in Results.

2. The results of the structural analysis are not analyzed critically. In particular, the authors did not present their full results for several parts of the results. Appropriate controls or comparisons should be made at every section to provide the reader with a better understanding of the strengths and limitations of the analysis (see main examples below).

We apologize for this, we have now written again different sections of the manuscript and parts of the discussion to make more clear the strengths and limitations.

3. The first part of the results is misleadingly called “structural analysis of the ACE2 - S protein complex”, while in fact it is a prediction of water molecules that are absent in selected structures the authors chose to analyze. I agree an analysis of waters can lead to new insights, but at the very least the authors need to apply it to all available structures and report the results in full and not in a qualitative and low resolution way for a selected (based on what exactly?) single structure, as in Fig. 1. For example, Fig. 1D does not really show in “atomic detail” the water networks. I believe it is critical to show which of these waters are predicted similarly in the majority of all comparable structures (including the Cov - 1 structures, but clearly stating which is which).

It is true that our message is not clear in Figure 1. We only wanted to point out that water bridges are very important in binding and that we made all our mutants and energy calculations

considering water molecules. Foldx has been shown to be very good in predicting crystallographic waters (Schymkowitz et al., 2005, 10.1073/pnas.0501980102). Following the reviewer's suggestion we have done a comparison of all crystal and predicted water bridges in the different structures availables. We find that we predict up to 100% of all crystal water bridges in the structures we have used. We have also compared the predicted waters for all the ACE/S protein complexes, we have visually found 9 tight clusters of waters that are common to all complexes in the binding surface of ACE2/S complex. We have modified Figure 1 to include the regions where the clusters lie as well as show the residues interacting with them (Figure 1B-D). We have also computed the contribution to binding energy of each water in the cluster using structure 6m0j and we provide the S6 Table with the coordinates of the predicted and crystallographic waters for 6m0j.

4. Recent papers have shown the assumptions made by the authors regarding infectivity in different species might be premature (e.g. dogs do seem to be susceptible). Better to analyze the bioinformatic data without assumptions, and leave the comparison to the literature for the Discussion.

We agree with the comment, we have changed the manuscript to show the comparison for the Discussion. The use of the new model (6m0j) that is much better (described in point 1) perfectly matches updated infectivities including dogs. We now included civet in the analysis of the species.

5. The in silico mutagenesis part is indeed important, but lacking. Why show as the main result here (Fig. 2A) a ddG per species? Much more interesting and useful to the community to show a per - residue plot for each species, aligned. Similarly, a full sequence alignment should be shown, rather that showing only selected residues (Fig. 2B).

We have modified the Figure 2 including full sequence alignment of ACE2 helix-cluster binding surface. And as the referee pointed out we have extracted the interacting residues defined as those that have a significant effect in binding energy upon mutation. We also calculate the stability change upon mutation for all of them in order to check for destabilization on ACE2 while doing mutagenesis. As the reviewer suggested, we now display the binding energies per species variant as a table in Fig 2 and the overall changes in binding energy compared to humans.

6. Certainly the authors can find much more detailed (cell - specific?) expression data to compare against their translation efficiency analysis? By now transcriptomic data is far from “scarce”.

The reviewer is right in mentioning that other sources of expression data are available. However, most of them cover only a few species and it is also not the intention of the manuscript to develop a full metadata analysis of transcriptomes. In fact, in the previous months, both bulk (Sun et al., 2020, 10.1101/2020.03.30.015644) and single-cell (Sungnak et al., 2020, 10.1038/s41591-020-0868-6) RNA sequencing datasets exploring the expression of ACE2 have already been published. Therefore, the novelty of the current manuscript does not lie in the analysis of ACE2 expression, but on the integration of both structural modeling and translational efficiency. All in all, single-cell sequencing datasets are only available for mice and humans (see Single-Cell Expression Atlas: ebi.ac.uk/gxa/sc/home), while data from bulk transcriptomics, despite covering more species, only very few of them cover upper respiratory tissues (see Bgee database: bgee.org). For all the reasons above, this manuscript includes only expression of lung tissues across species, which is the main tropism of SARS-CoV-2 and can be transversally evaluated for all animals except ducks and civets. We nevertheless acknowledge that the qualification of "scarce" is inappropriate, so now the sentence reads: "Although previous reports indicate that SARS-CoV-2 is also able to infect the upper respiratory tract in humans [23,26,37], gene expression data of that tissue is not available for most other species".

7. A critical analysis is missing – do mutate residues all along the spike protein to all possible residues, plot the predicted ddG values for stability and for the effect on interactions with ACE2, and also compare to known data on mutations in the spike protein(s) (i.e. why focus on ACE2 only). Even if prediction accuracy is far from perfect, such computational predictions can help scientists hypothesize what are the outcomes of mutations that are already being detected in the spike protein across the globe or that might be identified in the future.(No, Table S3 does not address this point at all.)

We performed the whole spike RBD mutational scanning using 2 models (6lzg, 6m0j), whose values are available in S4 Table. We computed protein S stability and binding affinity variations upon mutation in terms of free energy variation. We provide to the public a comprehensive list of predictions for protein S mutations and we present in the manuscript 20 mutations predicted to significantly increase the binding with ACE2 using the best available model (6m0j). We analysed the effect of mutating 6 residues recognized for being responsible for binding ACE2 receptors towards their homologous ones in SARS-CoV S protein. We further analysed our results and compared them with previous predictions from literature. We also compared our predictions with a list of 3773 observed missense variants of S protein, highlighting 6 significant amino acid mutations, all decreasing the interaction energy with human ACE2.

8. The discussion should also point where are the uncertainties in the predictions, and the known / predicted shortcomings of these predictions.

We based our analysis in the study of the point mutations effect on ACE2, and it is true that the cooperative effect of several mutations occurring in the same subject can be epistatic and induce conformational changes. This is one of the major limitations, in order to address this issue we have included an extra comparison between hACE2 and cACE2 to prove that the ACE2 binding surface constituted by 3 alpha helix (19-53, 54-83, 90-103) is highly conserved in the existing models (Fig 2, S1 Figure). We also discuss the example of the incorrectly placed side-chains of Asn/Gln in 6lzg, which results in the wrong prediction of D38E mutation. Finally, we discuss other issues like the energetic cost of cavities and the energy estimation for water bridges. All of this is included in a paragraph in the discussion.

9. A minor point – the M&M section should point to a supplementary document listing the full commands used to run the calculations. The current text is not sufficient to allow the reader to repeat the calculations as they were performed.

We apologize for this, we have written this section to make it more understandable to the reader and we have included a supplementary document (Supporting Information) explaining the commands required for the calculations.

Have all data underlying the figures and results presented in the manuscript been provided?

Large-scale datasets should be made available via a public repository as described in the *PLOS Computational Biology* data availability policy, and numerical data that underlies graphs or summary statistics should be provided in spreadsheet form as supporting information.

Reviewer #1: Yes

Reviewer #2: Yes
