# Supplementary material for: In silico mutagenesis of human ACE2 with S protein and translational efficiency explain SARS-CoV-2 infectivity in different species
Source: PLoS Comput Biol. 2020 Dec 7;16(12):e1008450. doi: 10.1371/journal.pcbi.1008450 (PMC7746295; doi:10.1371/journal.pcbi.1008450)
Supplement: Attachment — Submitted filename: Blanco_Serrano-PlosCB_review_Revision-20200901.pdf [file pcbi.1008450.s012.pdf]

While the authors revised the manuscript according to many of the minor comments, or minor parts of the major comments, the following major issues (and critically, in the Results section) have not been addressed:

1. The introduction was not expanded sufficiently to include an *appropriate* coverage of the *relevant* literature at this point in time. I repeat my strong recommendation to state the current time point at the end of the revision (e.g. "as of May 2020").

2. Some of the relevant structural papers are still not cited and previous research on SARS-CoV-1 or MERS vs. studies on the current SARS-CoV-2 was not clarified, emphasizing what is known about the different viruses/spike proteins and their common/different structural and sequence characteristics.

3. The results of the structural analysis have not been analyzed critically. Appropriate controls or comparisons were not added in a way that provides the reader with a better understanding of the strengths and especially the limitations of the analysis.

4. The first part of the results is still misleadingly called "structural *description* of the ACE2-S protein complex", while in fact it is *still* a prediction of water molecules that were predicted in the selected structures the authors chose to analyze. Fig. 1 is called "Structural description of the ACE2-S protein complex" while showing predicted waters. This is not reasonable! Why was this part not revised in a major way?

5. The in silico mutagenesis part (in my view the most important part of the manuscript) is still lacking. Why show as a main result a sum of values per species? This is not useful or detailed enough. Why did the authors not show a *full* (i.e. not just for selected residues) per-residue plot for each species, aligned – either as a graph or as a table? These are *the* main results of the whole manuscript, but they are not shown in full. (As an aside, raw results in Excel files that are not actually readable are not a reasonable way to show data, even in supporting information – do make the extra effort to make this data easily understandable by readers). This is a critical issue - the current figures do not show the important results in sufficient detail, at all.

6. The sudden move from structural analysis to translational efficiency and ACE2 expression lost me, as a reader. These parts do not connect at all (as rightly mentioned by Reviewer #1) and in the revised manuscript the disconnect is worse than in the original manuscript. The combination of the 2 parts is less than each separately, and I do not see how the separate parts of the Results combine to support the revised title of the manuscript. "In silico mutagenesis of human ACE2 with S protein and translational efficiency **explain SARS-CoV-2 infectivity** in different species"? No, the Results do not **explain** this… In fact, I second the suggestion made by Reviewer #1 to remove the translational efficiency part of the results entirely.

7. The critical analysis I mentioned previously is still missing – the authors did not show full in silico mutagenesis data all along the spike protein to all possible residues – plotting the predicted ddG values for stability and for the effect on interactions with ACE2, and also

comparing to known data on mutations in the spike protein(s). Yes, why not show the data for all structures rather than cherry pick one just because it has the best parameters? Even if prediction accuracy is far from perfect, showing the full results for multiple structures and both sides of the interface can help scientists hypothesize what are the outcomes of mutations that are already being detected in the spike protein across the globe or that might be identified in the future.

8. The discussion was not revised to point where are the uncertainties in the predictions, and the known / predicted shortcomings of these predictions.

9. The manuscript can benefit from English editing – in some cases I could not understand what the authors meant, while elsewhere non-scientific phrasing was used too often.
