# Supplementary material for: In silico mutagenesis of human ACE2 with S protein and translational efficiency explain SARS-CoV-2 infectivity in different species
Source: PLoS Comput Biol. 2020 Dec 7;16(12):e1008450. doi: 10.1371/journal.pcbi.1008450 (PMC7746295; doi:10.1371/journal.pcbi.1008450)
Supplement: Attachment — Submitted filename: Response_Letter_v2.pdf [file pcbi.1008450.s013.pdf]

Barcelona 08/10/2020

Dear Dr. Kolodny

Thanks for your mail. We have gone through the comments of reviewer 2 and we do not understand what the problem is. If you read all his/her comments, there are no formal issues with our work. Essentially everything resumes to format problems, and he/she does not point out to any fundamental flaw of our work. That is why we think the manuscript should be accepted. We could make new figures if needed, or change the way we present the data if you as Editor thinks it will make the manuscript stronger.

In the following lines, we will compare his/her previous comments and the new ones, and our response before and now. As you can see his/her are almost identical. Looking at what we wrote in the last version, to answer to his/her questions, you can see how we complied to what he/she asked for. All not critical data is in tables and we calculated energy changes for the three structures even if we mainly discuss one of them.

I think the reviewer wants a different type of paper where we compare different coronaviruses, but this is a different story, here we just wanted to explain why COVID-19 is very effective infecting humans compared to other species, and this is explained by structural analysis as well as by the codon adaptation index.

In any case, we have replied point by point to the reviewer and we have modified the manuscript where we think the referee has a point.

Thanks in advance

Luis Serrano

Comparative of comments between 2020-05-18 vs 2020-09-01

2020-05-18	2020-09-01
1. The introduction requires much improvement – it should be expanded and updated to include an appropriate coverage of the relevant literature at this point in time. Given how quickly the relevant literature is evolving, I strongly recommend stating the current time point at the end of the revision (e.g. "as of May 2020").<br><br>Some of the relevant structural papers are not cited (see also below). It is also important to clarify previous research on SARS-CoV-1 or MERS vs. studies on the current SARS-CoV-2, emphasizing what is known about the different viruses/spike proteins and their common/different structural and sequence characteristics. Such critical issues are not covered clearly nor comprehensively.	1. The introduction was not expanded sufficiently to include an *appropriate* coverage of the *relevant* literature at this point in time. I repeat my strong recommendation to state the current time point at the end of the revision (e.g. "as of May 2020").<br><br>2. Some of the relevant structural papers are still not cited and previous research on SARS-CoV-1 or MERS vs. studies on the current SARS-CoV-2 was not clarified, emphasizing what is known about the different viruses/spike proteins and their common/different structural and sequence characteristics.

Our reply to 2020-05-18:

We followed recommendations of the referee and apart from updating the literature we have made a thorough analysis of the most relevant crystal structures deposited in the protein data bank (PDB). Given the urgency this pandemic is causing we could have omitted this careful analysis so we thank the reviewer for pointing us in that direction. We were very surprised that some models with similar resolution don't have enough quality according to PDB sanity parameters (ramachandran, sidechain and RSRZ outliers) and FoldX energies, while others have suspiciously similar PDB coordinates that suggest that they were not assigned but created from other existing models. We have included a supplementary table (S5 Table) reporting quality parameters and per object RMSDs for the most important ACE2/SARS-CoV-2 S protein complexes. The best model according to these quality parameters and FoldX stability is 6m0j, which was not included in the previous version of the manuscript. For this reason, we have redone the same analysis with a new ACE2/SARS-CoV-2 S protein crystal structure (6m0j) that has passed all quality control filters and discarded 6vw1 which in fact is a chimaera and has low quality PDB parameters and bad packing energy compared to 6lzg and 6m0j. We have found, after looking carefully at 6lgz, that there is an intramolecular glutamine/asparagine network where the orientation of the side chain CO and NH2 groups are incorrectly placed (S2A-B Figure) which interacts with D38 of ACE2. This results in an incorrect prediction on binding energy when mutating D38 to Glu (see new Discussion). This network is in the correct orientation in 6m0j. Prediction of the binding energies in different species using 6m0j results in an excellent agreement with the experimental evidence (see new Figure 2). Changing the incorrect Gln network in 6lzg also results in the correct prediction of D38E mutation. We have included a block explaining the selection of the crystallographic models in Results.

GENERAL COMMENT:

This manuscript is not about a comparison of coronaviruses, it is about why the current coronavirus COVID19 infects some species and not others. The referee wants us to do a structural comparison of the spike proteins of different coronaviruses but this will be a different work. We think this requires an Editor's decision. Does the journal likes the concept of explaining the COVID-19 infectivity of different species?, if it is not the case then we should send the work to

another journal, we cannot do a detailed structural analysis on coronaviruses and at the same time address the COVID19 species specificity. As shown below we introduced a whole new paragraph in the intro with updated structural information on COVID-19.

New paragraph that was added to the Introduction in the first revision (changes of the second revision in italics):
Despite this rich interactome information and the existence of several viral structures, we only have structural information on the complex between the spike protein (S) and the host receptor angiotensin converting enzyme 2 (ACE2), which determines the cellular entry of the virus in the cell [13]. *Up to the time the manuscript was written (May 2020),* there is an electron microscopy structure of the complex of the SARS-CoV-2 S protein with the neutral amino acid transporter B0AT1 and the soluble part of human ACE2 (hACE2)[14]. There are are two crystal structures of the RBD domain of the S protein of SARS-CoV-2 and hACE2 (6moj [15]; 6lzg [16], as well as one of a chimeric RBD (SARS-CoV/SARS-CoV-2) with hACE2 (PDB id: 6vw1 [17]). Aside from these complexes, there is structural information of the interaction between the RBD domain of the S protein of other coronavirus and ACE2 from different hosts [16,18–20]. In these studies, it was mentioned that the major species barriers are determined by interactions between four ACE2 residues (residues 31, 35, 38, and 353) and two RBD residues (residues N479 and T487) [19]. Supporting the idea that the interaction of the S protein with ACE2 is critical for virus infection, it has been shown that, by changing four residues on the surface of rat ACE2 to human, rats can be infected by the SARS-CoV [21]. In this work, they identified residues 82-84 and 353 of ACE2 as critical for interaction with the S protein of this virus. Similarly, changing residues K479 and S487 in civet SARS-CoV S protein to N479 and T487 significantly enhanced the binding affinity for hACE2 [19].

2. The results of the structural analysis are not analyzed critically. In particular, the authors did not present their full results for several parts of the results. Appropriate controls or comparisons should be made at every section to provide the reader with a better understanding of the strengths and limitations of the analysis (see main examples below).	3. The results of the structural analysis have not been analyzed critically. Appropriate controls or comparisons were not added in a way that provides the reader with a better understanding of the strengths and especially the limitations of the analysis.

Our reply to 2020-05-18:

We apologize for this, we have now written again different sections of the manuscript and parts of the discussion to make more clear the strengths and limitations.

GENERAL COMMENT:

As you see from the parts of the Discussion we have added, I think we have discussed the limitations, like the quality of the structures etc…We cannot write a complete analysis on the problems and artifacts of protein design. There are numerous papers comparing the quality and success in predicting the effect of protein mutations, like the most recent one, showing that FoldX is among the top and indicating the main problems. We could add a reference to it:

Yang J, Naik N, Patel JS, Wylie CS, Gu W, Huang J, et al. (2020). Predicting the viability of beta-lactamase: How folding and binding free energies correlate with beta-lactamase fitness. PLoS ONE 15(5): e0233509. https://doi.org/10.1371/journal.pone.0233509

Paragraph that was added to the Results in the first revision discussing the limitations of the approach related to the quality of the structure:

Prior to any mutation modeling, we first looked at the PDB structure quality parameters (www.rcsb.org; S5 Table) to decide which structure is better for modelling. We found that 6m0j is the best by all criteria. This structure has only 0.1% of the residues in disallowed areas of the Ramachandran plot, followed by 6lzg that has 0.4% of disallowed residues. Structure 6vw1 aside from being a chimaera has 0% of the residues in disallowed areas but the lowest scores for the quality parameters. Thus we decided to use 6m0j as our structure to model the different variants in hACE2 and the RBD domain of the S protein (we also did the same mutations in 6lzg, see Discussion and Tables S1 to S5; with one exception discussed below there is an excellent correlation for the changes in energy upon mutation in both structures).

3. The first part of the results is misleadingly called "structural analysis of the ACE2-S protein complex", while in fact it is a prediction of water molecules that are absent in selected structures the authors chose to analyze. I agree an analysis of waters can lead to new insights, but at the very least the authors need to apply it to all available structures and report the results in full and not in a qualitative and low resolution way for a selected (based on what exactly?) single structure, as in Fig. 1. For example, Fig. 1D does not really show in "atomic detail" the water networks. I believe it is critical to show which of these waters are predicted similarly in the majority of all comparable structures (including the Cov-1 structures, but clearly stating which is which).	4. The first part of the results is still misleadingly called "structural *description* of the ACE2-S protein complex", while in fact it is *still* a prediction of water molecules that were predicted in the selected structures the authors chose to analyze. Fig. 1 is called "Structural description of the ACE2-S protein complex" while showing predicted waters. This is not reasonable! Why was this part not revised in a major way?

Our reply to 2020-05-18:

It is true that our message is not clear in Figure 1. We only wanted to point out that water bridges are very important in binding and that we made all our mutants and energy calculations considering water molecules. FoldX has been shown to be very good in predicting crystallographic waters (Schymkowitz et al., 2005, 10.1073/pnas.0501980102). Following the reviewer's suggestion we have done a comparison of all crystal and predicted water bridges in the different structures availables. We find that we predict up to 100% of all crystal water bridges in the structures we have used. We have also compared the predicted waters for all the ACE/S protein complexes, we have visually found 9 tight clusters of waters that are common to all complexes in the binding surface of ACE2/S complex. We have modified Figure 1 to include the regions where the clusters lie as well as show the residues interacting with them (Figure 1B-D). We have also computed the contribution to binding energy of each water in the cluster using structure 6m0j and we provide the S6 Table with the coordinates of the predicted and crystallographic waters for 6m0j.

GENERAL COMMENT:

We revised this in a major way, we did water prediction for the three available structures and we found 9 water clusters conserved in the three structures at the interface. We added a table with the coordinated of all predicted waters and we re-wrote this part. First, we say that a large part of the biding interface of the two proteins is dominated by waters, and that there is a large cavity between the two molecules. To make it a more throughout description of the interface, we have also included a description residue by residue of the interface, as the reviewer suggests.

**Description in the Results section (changes of the second revision in italics):**<br>hACE2 contacts the S protein through two separate regions leaving a central cavity that must be filled with water molecules (Fig 1A). *Using a simple 4.5Å contact distance cut-off, the residues involved on ACE2/S interface are hACE2-Q24 (sc-sc H-bond with S-N487), hACE2-T27 (hydrophobic packing with S-F456, S-Y473, S-A475, and S-Y480), hACE2-F28 (Van der Waals' contact with S-Y489), hACE2-D30 (sc-sc H-bond with S-K417), hACE2-K31 (hydrophobic packing with S-L455, S-F456, and S-Y489; salt bridge with S-E484; weak sc-sc h-bond S-Q493), hACE2-H34 (Van der Waals contact with S-Y453, and S-Q493), hACE2-E35 (sc-sc H-bond with S-Q493), hACE2-D38 (sc-sc H-bond with S-Y449), hACE2-Y41 (sc-sc H-bond with S-T500), hACE2-Q42 (sc-sc H-bond with S-Y449, and S-Q498; sc-mc h-bond with S-Y446), hACE2-L45 (hydrophobic packing with S-V445, and S-Q498), hACE2-L79 (hydrophobic packing with S-F486, and S-Y489), hACE2-M82 (hydrophobic packing with S-F486), hACE2-Y83 (sc-sc H-bond with S-N487; weak sc-sc H-bond with S-Y489; PI-PI interaction with S-F486), hACE2-Q325 (hydrophobic packing with S-V503), hACE2-N330 (Van der Waals contact with S-T500), hACE2-K353 (hydrophobic packing with S-Y505; sc-mc H-bond with S-G496), hACE2-D355 (weak sc-sc H-bond with S-T500), hACE2-D357 (weak sc-sc H-bond with S-T500) and hACE2-D393 (weak sc-sc H-bond with S-V503).* Water molecules bound at the interface of protein-protein interactions play an important role in affinity and specificity [29,30]. In fact, looking at the structure of the complex we find many instances of side chains from the two molecules capable of donating and/or accepting H-bonds that are close in space but not in contact (Fig 1B). These residues could interact via a water molecule (water bridge).

4. Recent papers have shown the assumptions made by the authors regarding infectivity in different species might be premature (e.g. dogs do seem to be susceptible). Better to analyze the bioinformatic data without assumptions, and leave the comparison to the literature for the Discussion.
**Our reply to 2020-05-18:**<br>*We agree with the comment, we have changed the manuscript to show the comparison for the Discussion. The use of the new model (6m0j) that is much better (described in point 1) perfectly matches updated infectivities including dogs. We now included civet in the analysis of the species.*

5. The in silico mutagenesis part is indeed important, but lacking. Why show as the main result here (Fig. 2A) a ddG per species? Much more interesting and useful to the community to show a per-residue plot for each species, aligned. Similarly, a full sequence alignment should be shown, rather that showing only selected residues (Fig. 2B).	5. The in silico mutagenesis part (in my view the most important part of the manuscript) is still lacking. Why show as a main result a sum of values per species? This is not useful or detailed enough. Why did the authors not show a *full* (i.e. not just for selected residues) per-residue plot for each species, aligned – either as a graph or as a table? These are *the* main results of the whole manuscript, but they are not shown in full. (As an aside, raw results in Excel files that are not actually readable are not a reasonable way to show data, even in supporting information – do make the extra effort to make this data easily understandable by readers). This is a critical issue - the current figures do not show the important results in sufficient detail, at all.

Our reply to 2020-05-18:

> *And as the referee pointed out we have extracted the interacting residues defined as those that have a significant effect in binding energy upon mutation. We also calculate the stability change upon mutation for all of them in order to check for destabilization on ACE2 while doing mutagenesis. As the reviewer suggested, we now display the binding energies per species variant as a table in Fig 2 and the overall changes in binding energy compared to humans.*

GENERAL COMMENT:

This referee's comment is a little bit vexing. In the new figure two, we added a panel with the contribution to free energy of individual residues at the interface of the two molecules that differ between the species (see Below Figure 2C). We did not add more residues because either they are identical in all species analyzed, or do not participate in the interaction. In any case all data is in an excel table in suppl material which I don't understand why it cannot be opened and read. All critical residues that are different between species are indicated and analyzed in this figure.

Figure of the first revision that showed the energy changes per position and variant:

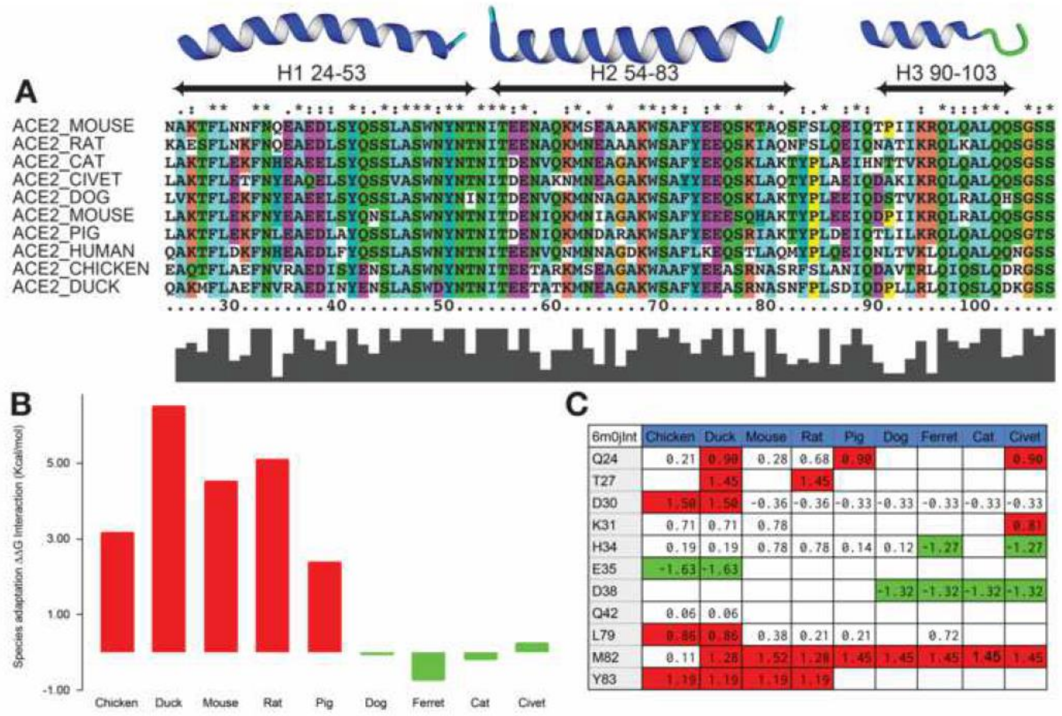

From Reviewer #1:	
3. The authors relate the infectivity of SARS-CoV-2 to the CAI of its genes in human and other potential hosts. They also attempt to draw a connection between ACE2 expression in host lung tissue and infectivity. It is noted that SARS-CoV-2 infects cats, yet the authors report some of the lowest CAIs for viral genes in Felis catus. They also report one of the lowest ACE2 expression levels in human lung. These contradictory analyses do not add to the message of the manuscript. CAI is a metric of	6. The sudden move from structural analysis to translational efficiency and ACE2 expression lost me, as a reader. These parts do not connect at all (as rightly mentioned by Reviewer #1) and in the revised manuscript the disconnect is worse than in the original manuscript. The combination of the 2 parts is less than each separately, and I do not see how the separate parts of the Results combine to support the revised title of the manuscript. "In silico mutagenesis of human ACE2 with S protein and translational efficiency explain SARS-CoV-2 infectivity in different species"?

codon usage bias and is not directly linked to translation efficiency. Additional approaches should be taken to estimate translation efficiency, otherwise this part should be eliminated altogether. The low expression of ACE2 in human lung and high expression in mouse lung (which is not infected by SARS-CoV-2) is not explained in the context of the study. In general, this is the weakest part of the study, it does not have novelty, while the conclusions are only loosely supported by the data.	No, the Results do not explain this... In fact, I second the suggestion made by Reviewer #1 to remove the translational efficiency part of the results entirely.

Our reply to 2020-05-18:

> *As suggested by the reviewer, we have now incorporated another well-established estimate of translational efficiency: the tRNA adaptation index (tAI). Rather than measuring the codon bias with regard to the host, the tAI shows the correspondence between the codon usage and the tRNA levels of the host (approximated by the tRNA gene copy numbers). Despite few differences, the tAI shows a very similar profile of adaptation across species (Fig. 3A-B). With this additional evidence, we believe that the effect of translational efficiency in determining viral infectivity is now more solid and stronger. Regarding the comments on the ACE2 gene expression, the data shown here indicates that low levels of ACE2 are sufficient to cause infection, as far as the virus has an optimal interaction with the spike and is translationally adapted. In fact,* Homo sapiens *is the species with both the highest tAI and the highest interaction, which could explain the high transmissibility of the virus among humans.*

GENERAL COMMENT:

I think we made it clear why we included the CAI and tAI in the paper. Based on structures we cannot say why the COVID-19 preferentially infects humans over cats or ferrets. The CAI/tAI shows a very good adaptation of COVID-19 to humans and poor to the other species that structurally should be similar as humans. Thus, we suggest that aside having a good energy of interaction between virus and host proteins translational efficiency of the viral genes is important. We have added a new sentence in the Results section to make it clearer now. If the Editor thinks that we should remove this section, we could, but we remove a good argument worth considering regarding species infectivity, which is the main story line of the manuscript, not the comparison of different coronavirus.

Explanations in the first revision of why we include this analysis (changes of the second revision in italics):

Introduction

A second factor that is important for virus infectivity is the adaptation of the viral codon usage to that of its host [22,23]. The universal genetic code indicates that multiple 3- letter combinations of nucleotides can encode for the same amino acid (aka synonymous codons). However, these different synonymous codons can be recognized distinctly by cellular tRNAs, leading to differences in translational efficiency [24]. In particular, in terms of translational adaptation of SARS-CoV-2 to human tissues, the viral proteome is especially adapted to the tRNA levels of the upper respiratory tract and the lung parenchyma [23]. This is also in agreement with single-cell transcriptomics describing ACE2 expression in nasal goblet and ciliated cells as well as type-2 alveolar epithelial cells [25,26]. In concordance, patients of SARS-81 CoV-2 showcase high viral loads in nasal swabs compared to other tissues of the respiratory tract [27].

Here, we have analyzed two of factors that could affect the infectivity of the SARS-CoV-2 in different species as well as the possible sensitivity of humans with different ACE2 variants. […]

Second, we found out the high translational adaptation of SARS-CoV-2 in *Homo sapiens*, compared to other species, which could explain its high infectivity in humans.

Results

While the infectivity of different species can be explained by the ACE2-S protein binding affinity, this alone cannot completely explain the severity of the disease in each species. Upon the productive interaction of the viral spike glycoprotein and the cell receptor ACE2, the viral genome enters the cell and starts its replication. The coronavirus therefore needs to hijack the translational machinery of the host to efficiently replicate and produce new virions. In this context, the codon usage of viral proteins should potentially resemble that of the host cell in order to adapt to the tRNA pools that drive an optimal translation [23].

[…] CAI and tAI as estimates of translational efficiency could better explain, together with binding affinity, species infectivity. We observed that humans are the species in which SARS-CoV-2 is both most efficiently translated as well as optimally interacting with ACE2.

7. A critical analysis is missing – do mutate residues all along the spike protein to all possible residues, plot the predicted ddG values for stability and for the effect on interactions with ACE2, and also compare to known data on mutations in the spike protein(s) (i.e. why focus on ACE2 only). Even if prediction accuracy is far from perfect, such computational predictions can help scientists hypothesize what are the outcomes of mutations that are already being detected in the spike protein across the globe or that might be identified in the future.(No, Table S3 does not address this point at all.)	7. The critical analysis I mentioned previously is still missing – the authors did not show full in silico mutagenesis data all along the spike protein to all possible residues – plotting the predicted ddG values for stability and for the effect on interactions with ACE2, and also

Our reply to 2020-05-18:

> *We performed the whole spike RBD mutational scanning using 2 models (6lzg, 6m0j), whose values are available in S4 Table. We computed protein S stability and binding affinity variations upon mutation in terms of free energy variation. We provide to the public a comprehensive list of predictions for protein S mutations and we present in the manuscript 20 mutations predicted to significantly increase the binding with ACE2 using the best available model (6m0j). We analyzed the effect of mutating 6 residues recognized for being responsible for binding ACE2 receptors towards their homologous ones in SARS-CoV S protein. We further analysed our results and compared them with previous predictions from literature. We also compared our predictions with a list of 3773 observed missense variants of S protein, highlighting 6 significant amino acid mutations, all decreasing the interaction energy with human ACE2.*

GENERAL COMMENT:

We did what the referee asked and we put all data on the excel tables in suppl material and in the text we mentioned the ones that were important. How we could show in a figure hundreds of positions and thousands of mutations affecting stability? If the editor thinks this is important we could try, but it will be a kind of gene heatmap with hundreds of positions that will not allow anyone to get any useful information.

Explanations in the first revision (changes of the second revision in italics):

ACE2 human variation and interaction with S protein

We found a total of 260 reported single point mutations (S3 Table, which also includes allelic frequencies). We mutated each of the positions in 6moj using FoldX and determined the changes

in stability of the ACE2 protein, as well as in interaction energy with the S protein (S3 Table) for mutations having significant effects, with more than 0.8 kcal/mol in absolute terms [39]. We find only one variant G326E that significantly improves binding energy without destabilizing the hACE2 protein (Fig 4 and S3 Table). There are two mutations that consistently decrease binding without affecting hACE2 stability and could confer protection (E37K, T27A). We also found that 110 out of 260 mutations destabilize significantly the ACE2 protein (>1.5 kcal/mol), which could prevent its correct folding and therefore the binding to the virus (S3 Table).<br>***S protein point mutation energy landscape and interaction with ACE2***<br>Using the 6m0j structure, we generated each of the 20 possible mutations for all RBD residues (S4 Table). The percentage of mutations that destabilize the spike protein is ~43%, while ~1% stabilizes it. Considering the interface residues of the complex, we found that ~35% of the mutations would decrease binding affinity of the complex and only ~6% of them would improve it.<br>We observed that several mutations on five interface residues (V445M, V445R, V445W; Q493F, Q493L, Q493M, Q493Y; Q498F, Q498L, Q498M, Q498Y; T500K; N501A, N501C, N501L, N501S, N501T, V503R, 503W, V503Y) were beneficial for the interaction with hACE2 without destabilizing the S domain. Based on the coding sequence of S protein, we observe that, out of the 20 favourable amino acid substitutions, 4 would require at least one nucleotide mutation, 9 at least two mutations, and the remaining 7 three nucleotide mutations. We compared our mutational interaction energy landscape of S protein with a list of 3773 observed missense variants resulting in 2420 unique amino acid mutations found in S protein gene using the CRG Viral Beacon (covid19beacon.crg.eu). Among the observed natural mutations we detected 6 of them predicted as detrimental for the interaction with hACE2: L455F, A475V, K417N, N487K, Y489H, A475S (S4 Table) and we did not observe any of the ones that improve binding.<br>Residues Q493 and N501 belong to a group of six interface residues (L455, F486, Q493, S494, N501, Y505) fundamental for binding to ACE2 receptors and for determining the host range of SARS-CoV-like viruses [3]. Although the effect of multiple mutations on interaction energy is not necessarily additive when making a single multiple mutant, we observe that mutating the 6 positions mentioned to their relative ones in SARS-CoV S protein lowers the interaction affinity by +5.2 kcal/mol in 6m0j. Interestingly, residue N501 corresponds to a Thr in SARS-CoV S protein and we find that, in agreement with previous predictions [37], mutating N501 to Thr improves binding to hACE2 (S4 Table).

8. The discussion should also point where are the uncertainties in the predictions, and the known / predicted shortcomings of these predictions.	8. The discussion was not revised to point where are the uncertainties in the predictions, and the known / predicted shortcomings of these predictions.
**Our reply to 2020-05-18:**<br>*We based our analysis in the study of the point mutations effect on ACE2, and it is true that the cooperative effect of several mutations occurring in the same subject can be epistatic and induce conformational changes. This is one of the major limitations, in order to address this issue we have included an extra comparison between hACE2 and cACE2 to prove that the ACE2 binding surface constituted by 3 alpha helix (19-53, 54-83, 90-103) is highly conserved in the existing models (Fig 2, S1 Figure). We also discuss the example of the incorrectly placed side-chains of Asn/Gln in 6lzg, which results in the wrong prediction of D38E mutation. Finally, we discuss other issues like the energetic cost of cavities and the energy estimation for water bridges. All of this is included in a paragraph in the discussion.*<br>**GENERAL COMMENT:**	

As I mentioned above, we introduced a paragraph in the Discussion pointing to the uncertainties, we cannot write a paper on the problems of protein design here.

Discussion paragraph in the first revision:
Although, we see an excellent correlation between the effect of mutating residues of hACE2 in the 6moj and 6lzg structures there is one case where the result is very different. This happens at position D38 where mutation to Glu is favourable in 6m0j and unfavourable in 6lzg (S4 Table). The reason for this difference is the network of H-bonds made by the side chains of hACE2 Q42, Q498 and Y449, which is incorrect in 6lzg. The reason is that in the 6lzg structure the proton of the OH side chain group of Y449 (S protein) is donated to the hACE2 295 D38 carboxylate group, as a result the O of the Tyr449 OH group is at H-bond distance of the oxygen of the CO side chain group of Q498 (hACE2) which is not possible (Fig S1C-D). FoldX cannot repair this because there is a double reciprocal H bond between the side chains of Q42 and Q498 in hACE2 and therefore it does not move them. This does not happen in 6m0j where all H-bonds are correct and allow substitution of D38 by Glu without destabilizing the complex. Thus it is important to examine the quality of the structures prior to the *in silico* mutagenesis. In any case, and for information purposes, we include the same data presented in this work for 6m0j and for 6lzg which are in very good agreement except for a few cases as the one mentioned here (S1 Table and S4 Table).

9. A minor point – the M&M section should point to a supplementary document listing the full commands used to run the calculations. The current text is not sufficient to allow the reader to repeat the calculations as they were performed.	9. The manuscript can benefit from English editing – in some cases I could not understand what the authors meant, while elsewhere non-scientific phrasing was used too often.

Our reply to 2020-05-18:

We apologize for this, we have written this section to make it more understandable to the reader and we have included a supplementary document (Supporting Information) explaining the commands required for the calculations.

GENERAL COMMENT:

The manuscript was read by a native speaker, but we can send it to a professional.

Reviewer 2

Thanks for your comments, below you will find our reply to them.

1. **The introduction was not expanded sufficiently to include an appropriate coverage of the relevant literature at this point in time. I repeat my strong recommendation to state the current time point at the end of the revision (e.g. "as of May 2020").**

The reviewer is right that in a fast-moving field like this one we need to indicate the current time at which we did our study. This is now included in the new version.

2. **Some of the relevant structural papers are still not cited and previous research on SARS-CoV-1 or MERS vs. studies on the current SARS-CoV-2 was not clarified, emphasizing what is known about the different viruses/spike proteins and their common/different structural and sequence characteristics.**

We have cited all relevant structures to the SARS-CoV-2, we have not include all papers dealing with SARS-CoV-1 and other coronavirus since we don't think they are relevant to our paper that is focused on the SARS-CoV-2 and its interaction withhACE2. What the reviewer proposes will be a different manuscript that will be a comparative structural analysis of coronavirus spike proteins.

3. **The results of the structural analysis have not been analyzed critically. Appropriate controls or comparisons were not added in a way that provides the reader with a better understanding of the strengths and especially the limitations of the analysis.**

As you see from the parts of the Discussion we added in the first revision, we think we have discussed the limitations, like the quality of the structures etc… We cannot write a complete analysis on the problems and artifacts of protein design. There are numerous papers comparing the quality and success in predicting the effect of protein mutations, like the most recent one, showing that FoldX is among the top force fields and indicating the main problems:

Yang J, Naik N, Patel JS, Wylie CS, Gu W, Huang J, et al. (2020). Predicting the viability of beta-lactamase: How folding and binding free energies correlate with beta-lactamase fitness. PLoS ONE 15(5): e0233509. https://doi.org/10.1371/journal.pone.0233509

4. **The first part of the results is still misleadingly called "structural description of the ACE2-S protein complex", while in fact it is still a prediction of water molecules that were predicted in the selected structures the authors chose to analyze. Fig. 1 is called "Structural description of the ACE2-S protein complex" while showing predicted waters. This is not reasonable! Why was this part not revised in a major way?**

We only wanted to point out that water bridges are very important in binding and that we made all our mutants and energy calculations considering water molecules. Following the referee's advice, we have included a full description of the residues involved on the interface and the kind of interaction that can be formed with the S protein. Now it reads (changes in italics): "hACE2 contacts the S protein through two separate regions leaving a central cavity that must be filled with water molecules (Fig 1A). *Using a simple 4.5Å contact distance cut-off, the residues involved on ACE2/S interface are hACE2-Q24 (sc-sc H-bond with S-N487), hACE2-T27 (hydrophobic packing with S-F456, S-Y473, S-A475, and S-Y480), hACE2-F28 (Van der Waals' contact with S-Y489), hACE2-D30 (sc-sc H-bond with S-K417), hACE2-K31 (hydrophobic packing with S-L455, S-F456, and S-Y489; salt bridge with S-E484; weak sc-sc h-bond S-Q493), hACE2-H34 (Van der Waals contact with S-Y453, and S-Q493), hACE2-E35 (sc-sc H-bond with S-Q493), hACE2-D38 (sc-sc H-bond with S-Y449), hACE2-Y41 (sc-sc H-bond with S-T500), hACE2-Q42 (sc-sc H-bond with S-Y449, and S-Q498; sc-mc h-bond with S-Y446), hACE2-L45 (hydrophobic packing with S-V445, and S-Q498), hACE2-L79 (hydrophobic packing with S-F486, and S-Y489), hACE2-M82 (hydrophobic packing with S-F486), hACE2-Y83 (sc-sc H-bond with S-N487; weak sc-sc H-bond with S-Y489; PI-PI interaction with S-F486), hACE2-Q325 (hydrophobic packing with S-V503), hACE2-N330 (Van der*

Waals contact with S-T500), hACE2-K353 (hydrophobic packing with S-Y505; sc-mc H-bond with S-G496), hACE2-D355 (weak sc-sc H-bond with S-T500), hACE2-D357 (weak sc-sc H-bond with S-T500) and hACE2-D393 (weak sc-sc H-bond with S-V503). Water molecules bound at the interface of protein-protein interactions play an important role in affinity and specificity [29,30]. In fact, looking at the structure of the complex we find many instances of side chains from the two molecules capable of donating and/or accepting H-bonds that are close in space but not in contact (Fig 1B). These residues could interact via a water molecule (water bridge)".

5. **The in silico mutagenesis part (in my view the most important part of the manuscript) is still lacking. Why show as a main result a sum of values per species? This is not useful or detailed enough. Why did the authors not show a full (i.e. not just for selected residues) per-residue plot for each species, aligned – either as a graph or as a table? These are the main results of the whole manuscript, but they are not shown in full. (As an aside, raw results in Excel files that are not actually readable are not a reasonable way to show data, even in supporting information – do make the extra effort to make this data easily understandable by readers). This is a critical issue - the current figures do not show the important results in sufficient detail, at all.**

In the new figure two, we added a panel with the contribution to free energy of individual residues at the interface of the two molecules that differ between the species (see below Figure 2C). We did not add more residues because either they are identical in all species analyzed, or do not participate in the interaction. It does not make sense to represent hundreds of residues if they are not involved in the interaction between hACE2 and the spike protein or if they are identical in all species. In any case, all data is in an excel table in suppl material. All critical residues that are different between species are indicated and analyzed in this figure.

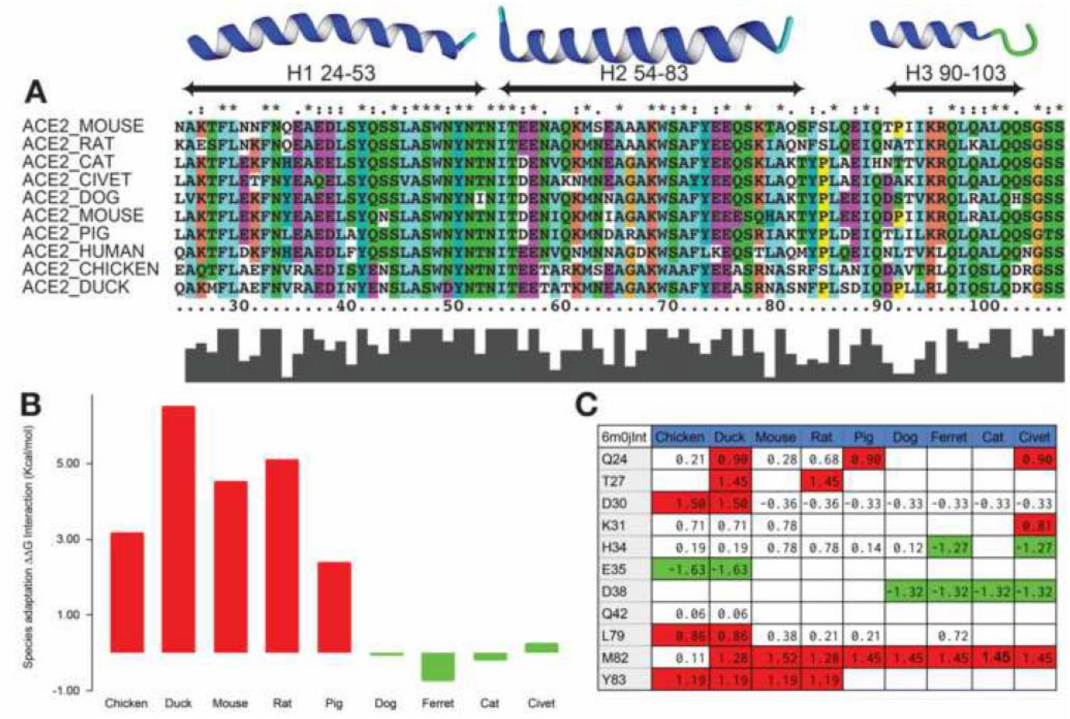

6. **The sudden move from structural analysis to translational efficiency and ACE2 expression lost me, as a reader. These parts do not connect at all (as rightly mentioned by Reviewer #1) and in the revised manuscript the disconnect is worse than in the original manuscript. The combination of the 2 parts is less than each separately, and I do not see how the separate parts of the Results combine to support the revised title of the manuscript. "In silico mutagenesis of human ACE2 with S protein and translational efficiency explain**

SARS-CoV-2 infectivity in different species"? No, the Results do not explain this… In fact, I second the suggestion made by Reviewer #1 to remove the translational efficiency part of the results entirely.

As mentioned to Reviewer #1 in the first revision, based on structures alone we cannot tell why the COVID-19 preferentially infects humans over cats or ferrets. Both the CAI and tAI show a very good adaptation of COVID-19 to humans, but poor to the other species that should be structurally similar to humans. Thus, we suggest that, apart from having a good energy of interaction between virus and host, translational efficiency of the viral genes can play an important role. Although Reviewer #1 has already confirmed that his concerns on the issue had already been satisfied, we have included an additional sentence at the beginning of this section to further clarify its connection with the rest of the article: "While the infectivity of different species can be explained by the ACE2-S protein binding affinity, this alone cannot completely explain the severity of the disease in each species".

7. **The critical analysis I mentioned previously is still missing – the authors did not show full in silico mutagenesis data all along the spike protein to all possible residues – plotting the predicted ddG values for stability and for the effect on interactions with ACE2, and also comparing to known data on mutations in the spike protein(s). Yes, why not show the data for all structures rather than cherry pick one just because it has the best parameters? Even if prediction accuracy is far from perfect, showing the full results for multiple structures and both sides of the interface can help scientists hypothesize what are the outcomes of mutations that are already being detected in the spike protein across the globe or that might be identified in the future.**

Since our first revision we performed the whole spike RBD mutational scanning with ΔΔG prediction using 2 models (6lzg, 6m0j) as requested by the reviewer. The analysis is clearly explained in the manuscript and data are available in S4 Table. We computed protein S stability and binding affinity variations upon mutation in terms of free energy variation. We provide to the public a comprehensive list of predictions for protein S mutations and we present in the manuscript 20 mutations predicted to significantly increase the binding with ACE2 using the best available model (6m0j). In fact, we analyzed the effect of mutating 6 residues known to be responsible for binding ACE2 receptors towards their homologous ones in SARS-CoV S protein. We further analysed our results and compared them with previous predictions from literature. We also compared our predictions with a list of 3773 observed missense variants of S protein, highlighting 6 significant amino acid mutations, all found decreasing the interaction energy with human ACE2.

8. **The discussion was not revised to point where are the uncertainties in the predictions, and the known /predicted shortcomings of these predictions.**

We based our analysis on the study of the point mutations effect on ACE2, and it is true that the cooperative effect of several mutations occurring in the same subject can be epistatic and induce conformational changes. This is one of the major limitations, in order to address this issue we have included an extra comparison between hACE2 and cACE2 to prove that the ACE2 binding surface constituted by 3 alpha helix (19-53, 54-83, 90-103) is highly conserved in the existing models (Fig 2, S1 Figure). We also discuss the example of the incorrectly placed side-chains of Asn/Gln in 6lzg, which results in the wrong prediction of D38E mutation. Finally, we discuss other issues like the energetic cost of cavities and the energy estimation for water bridges. All of this was included in a paragraph in the discussion.

9. **The manuscript can benefit from English editing – in some cases I could not understand what the authors meant, while elsewhere non-scientific phrasing was used too often.**

A native speaker read the manuscript, but we can send it to a professional.
